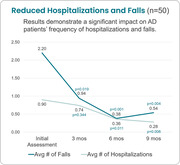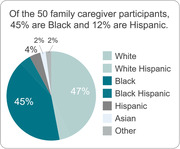# Virtual support for family caregivers to reduce hospitalization and fall risk in people with Alzheimer’s disease

**DOI:** 10.1002/alz.094318

**Published:** 2025-01-09

**Authors:** Rosemary D. Laird, Jason Abrevaya

**Affiliations:** ^1^ Navigating Aging Needs, LLC, Melbourne, FL USA; ^2^ University of Texas‐Austin, Austin, TX USA

## Abstract

**Background:**

The Florida Department of Elder Affairs and Navigating Aging Needs, LLC (NAN) recently formed a public‐private partnership providing virtual support to family caregivers of people with Alzheimer’s disease (AD) living at home. The program targets a diverse population with high‐level daily care needs and at increased risk for continuing decline and costly Medicaid‐supported care. Objectives To reduce the risk of hospitalizations and falls for people living with Alzheimer’s disease.

**Methods:**

Family caregivers were offered a virtual support program that included regular meetings (Zoom or phone) with a personal licensed social worker. Of the 50 family caregiver participants, 45% were Black and 12% were Hispanic. The initial assessment included 80 questions on the AD patient’s medical, emotional, social, and legal/financial well‐being and the validated battery of 12 questions comprising the Zarit Burden Scale. Following the assessment, the social worker provided the family caregiver with a personal plan that identified areas of risk that “need attention,” along with resources to address identified needs. The social worker and family caregiver then met monthly to resolve areas identified as high risk and discuss other relevant issues as they arose. Every 3 months the social worker re‐assessed patient status with the family caregiver.

**Results:**

After 9 months of virtual support, family caregivers reported 75% fewer falls and 68% fewer hospitalizations. Average number of falls fell from 2.2 at baseline to 0.54 (p = 0.004), and average number of hospitalizations fell from 0.90 at baseline to 0.28 (p = 0.006). Caregiver assessments also demonstrated a positive impact on AD patients' depression, anxiety, advanced directives, and financial planning over the course of the program. Zarit Burden Scale scores for family caregivers remained stable despite AD progression.

**Conclusion:**

Providing virtual support to family caregivers of people with AD was correlated with reduced hospitalizations and falls for people living at home with AD in this pilot program. The next step for the Florida Department of Elder Affairs and NAN is to assess the cost implications of expanding this approach on a larger scale.